# Optimized ex vivo stimulation identifies multi-functional HBV-specific T cells in a majority of chronic hepatitis B patients

**DOI:** 10.1038/s41598-020-68226-5

**Published:** 2020-07-09

**Authors:** Conan G. Chua, Aman Mehrotra, Tony Mazzulli, David K. Wong, Jordan J. Feld, Harry L. A. Janssen, Adam J. Gehring

**Affiliations:** 1 Toronto Centre for Liver Disease, Toronto General Hospital Research Institute, University Health Network, Toronto, Canada; 2 Institute of Medical Sciences, University of Toronto, Toronto, Canada; 3 Department of Laboratory Medicine and Pathobiology, University of Toronto, Toronto, Canada; 4 Department of Microbiology, Mount Sinai Hospital, University Health Network, Toronto, Canada

**Keywords:** Lymphocyte activation, Immunology, Hepatology

## Abstract

High antigen burden during chronic hepatitis B (CHB) results in a low frequency HBV-specific T cell response with restricted functionality. However, this observation is based on limited data because low T cell frequencies have hindered effective ex vivo analysis. We adapted the ELISpot assay to overcome this obstacle to measure ex vivo T cell responses in CHB patients. We modified the key variables of cell number and the peptide pulsing method to improve ex vivo detection of HBV-specific T cells. We detected IFN-γ responses in 10/15 vaccinated controls and 20/30 CHB patients, averaging 195 and 84 SFUs/2 × 10^6^ PBMCs respectively. Multi-analyte FluoroSpots improved functional characterization of T cells. We detected IFN-γ responses in all tested vaccinated controls (n = 10) and CHB patients (n = 13). IL-2 responses were detectable in 9/10 controls and 10/13 patients. TNF-α displayed less sensitivity, detectable in only 7/10 controls and 7/13 patients. Antigen-specific analysis demonstrated that IFN-γ responses were dominated by polymerase and core, with weak responses to envelope and X. IL-2 responses were found in 3/5 patients and equally directed towards polymerase and core. While their ex vivo frequency is extremely low, a fraction of HBV-specific T cells are detectable and display multi-functionality ex vivo.

## Introduction

Over 250 million individuals worldwide are chronically infected with Hepatitis B virus (HBV)^1^, which causes more than 700,000 deaths annually and is the primary risk factor for liver cancer^2,3^. The increasing recognition of this public health issue has accelerated research into new treatments to cure chronic hepatitis B (CHB), many of which are directed at restoring antiviral immunity to control HBV infection.

New immunotherapeutic strategies target T cells because of their critical role in resolving HBV infection^4^. Patients, who clear chronic HBV infection after long-term antiviral therapy display a T cell repertoire with an expansion capacity similar to resolved individuals^5^. However, virus-specific T cell responses in CHB patients are quantitatively weak^6,7^. The low frequency has been the primary obstacle to accurately defining both the ex vivo phenotype and functionality of HBV-specific T cells. To overcome the low frequency, adaptations of conventional immunological assays are required. Tetramer enrichment pulls HBV-specific T cells out of the tens of millions of cells that would have to be analyzed to obtain a significant number of events for robust analysis. This adaptation to tetramer staining improved the ex vivo phenotypic understanding of T cells targeting different HBV antigens^8,9^.

Similar obstacles have impeded our understanding of HBV-specific T cell functionality ex vivo. The inability to consistently measure the functionality of HBV-specific T cells ex vivo has prevented meaningful analysis during antiviral events such as HBeAg loss, HBsAg loss and hepatic flares^10^. The lack of functional detection has also impeded our understanding of the immunological effects of both new and existing therapies^10–13^. Recognizing this, the International Coalition to Eliminate HBV (ICE-HBV) called for development of more sensitive assays to measure HBV immunity ex vivo^14^.

Similar to the tetramer enrichment strategy, we adapted conventional ELISpot-based assays to accurately measure virus-specific T cell functionality ex vivo. We identified two key weaknesses with established approaches: (1) low frequencies of HBV-specific T cells fall beneath the lower limit-of-detection in conventional ELISpots and (2) using individual pools for each HBV protein will exclude antigen-specific responses—i.e. HBV core antigen (HBcAg)-specific T cells will not be activated in HBV surface antigen (HBsAg) stimulated wells. Our adapted assay detected ex vivo HBV-specific T cell responses in 67% of CHB patients, allowing us to correlate functional T cell frequency with different clinical and viral biomarkers. Translating our strategy to a 3-color FluoroSpot assay improved ex vivo detection and identified the presence of multi-functional T cells displaying an HBV antigen hierarchy consistent with previous studies^8,9^. By adapting the conventional ELISpot, we provide the first robust measurement of HBV-specific T cell functionality directly ex vivo in CHB patients. This assay will help define the potential for virus-specific T cell functionality to serve as an immunological biomarker of HBV control.

## Results

### *Optimization of *ex vivo* IFN-y ELISpot parameters*

**Figure 1:**
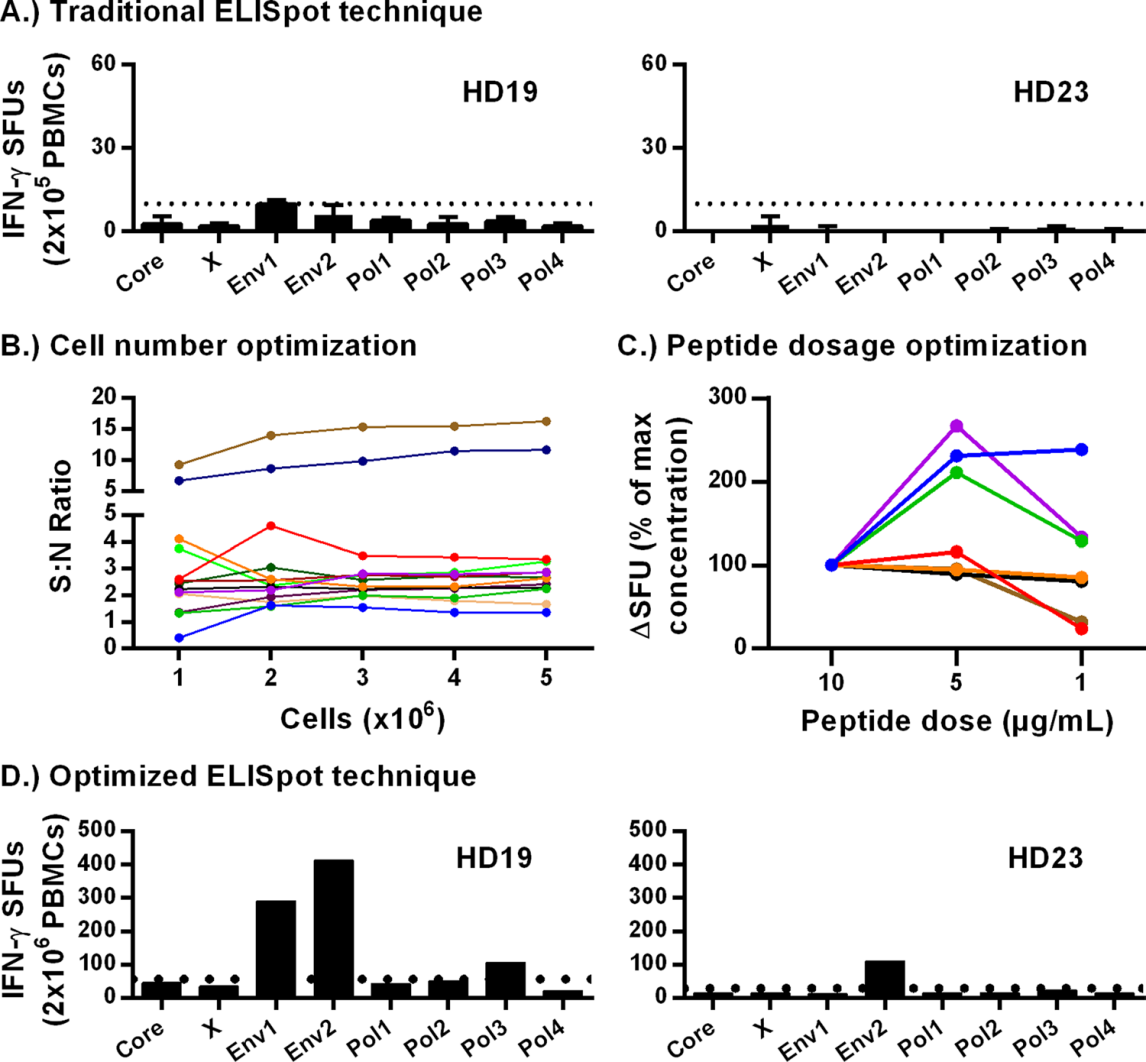
Optimization and improvement of ex vivo ELISpot assay. (**A**) Triplicates of 2 × 10^5^ vaccinated donor PBMCs (n = 10) were stimulated at 5 µg/mL/peptide with respective OLP pools. (**B**) 1–5 × 10^6^ vaccinated donor PBMCs (n = 13) were plated and simultaneously pulsed at 10 µg/mL/peptide with all OLP pools. Signal-to-noise ratios were calculated. (**C**) 2 × 10^6^ vaccinated donor PBMCs (n = 7) were stimulated with 10, 5, and 1 µg/mL/OLP. (**D**) 2 × 10^6^ vaccinated donor PBMCs (n = 10) were pulsed at 5 µg/mL/peptide with OLP pools (8) for each HBV antigen. Dotted lines indicate respective positive thresholds.

To identify the limitations of the standard IFN-γ ELISpot approach in detecting HBV-specific T cell responses, we measured HBV-specific T cells directly ex vivo in healthy vaccinated donors. The commercial HBV vaccines consist of the HBV surface antigen (HBsAg) in alum. Therefore, envelope (HBsAg)-specific CD4 T cells should be present in vaccinated donors. All vaccinated donors had a confirmed anti-HBs titre (not shown). Vaccinated donor PBMCs (n = 10) were thawed, and rested overnight. PBMCs were plated in triplicate at 2 × 10^5^ cells/well in Aim-V without serum and stimulated with individual synthetic over-lapping peptide (OLP) pools covering each HBV protein: 1 core (C), 1 X, 2 envelope (Env), 4 polymerase (Pol) and incubated at 37 °C in 5% CO_2_ for 24 h. Responses were positive if the average of triplicate wells exceeded 10 SFUs and was twice the mean of DMSO-control wells. Detectable HBV-specific T cell responses were seen in only 4/10 of vaccinated donors using the standard approach to IFN-γ ELISpot analysis (Fig. 1A; Supplementary Fig. S1A). In addition, the magnitude of positive responses was low, with fewer than 60 spots in three of the responding donors. As anticipated, responses were specific to the HBV envelope protein. Therefore, even using vaccinated donors that were expected to have an HBV antigen-specific response, we had difficulty detecting HBV-specific T cells above the positive cutoff threshold ex vivo.

We hypothesized that majority of vaccinated donors should have an HBV envelope-specific T cell response. However, 10 SFUs in 2 × 10^5^ PBMCs is equivalent to a peripheral frequency of 0.005% (1:20,000). We believed that this low frequency prevents consistent detection with current iterations of the IFN-γ ELISpot. Therefore, we approached this problem as a mathematical limitation of T cell frequency. In addition, vaccine (envelop)-specific T cells could be split between any of the eight peptide pools, meaning a fraction will not receive stimulation and thus will not be detected. This practical issue further hinders the detection of HBV-specific responses. Thus, we predicted that plating more PBMCs and pulsing with all HBV OLP pools simultaneously will significantly improve the detection of total HBV-specific T cell responses and overcome the mathematical limitation of low T cell frequencies.

**Figure 2:**
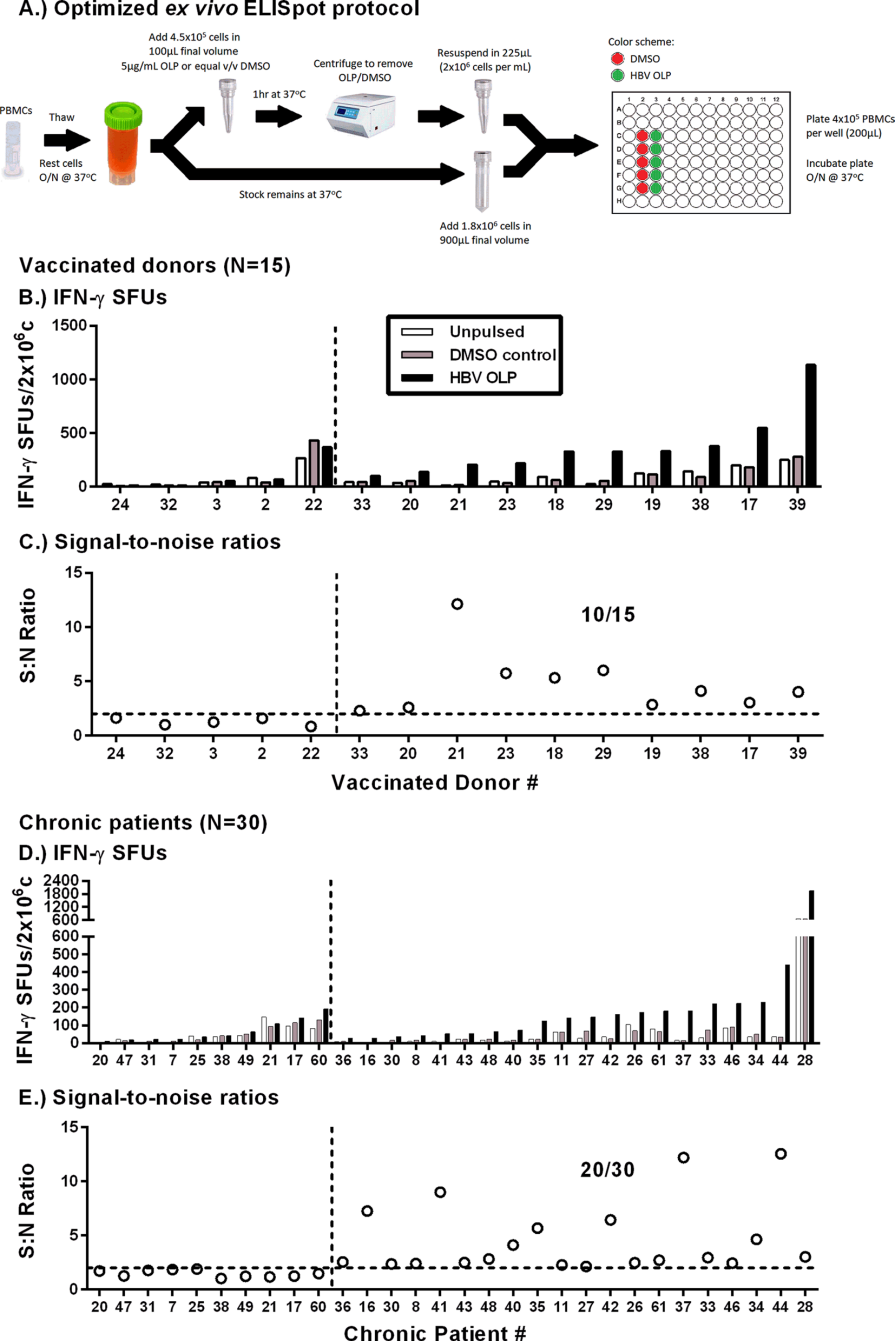
67% of chronic patients had detectable HBV-specific T cells ex vivo. (**A**) Schematic of the optimized peptide pulsing and ELIspot plating strategy. The same peptide pulsing and plating strategy was used for the Fluorospot assay and single OLP pool stimulations to determine antigen-specificity (i.e. Figs. 1D, 6B). (**B**) Absolute SFU and (**C**) S:N ratios of 15 vaccinated donors tested with ex vivo IFN-γ ELISpot. (**D**) Absolute SFU and (**E**) S:N ratios of 30 CHB patients. To the left of the vertical dotted lines are non-responders. To the right are ELISpot-positive responders. Horizontal dotted lines indicate the positive S:N ratio threshold of 2.

To test whether increasing the number of PBMCs overcomes the mathematical limitation of antigen-specific cells, and whether changing the peptide pulsing strategy increases quantifiable SFUs, we stimulated up to 5 × 10^6^ PBMCs from vaccinated donors (n = 10) with all 8 peptide pools. To minimize PBMC exposure to DMSO, we adapted a peptide stimulation strategy used to successfully expand HBV-specific T cells from CHB patient in vitro^15^. Using this strategy, only 20% of the PBMC are exposed to peptides and DMSO vehicle, while the remaining 80% are left in culture medium until being combined in the ELISpot plate (Fig. 2A). Stimulated PBMCs were then plated at 2 × 10^5^ or 4 × 10^5^ cells/well. To effectively count 10^6^ cells, two wells of 4 × 10^5^ and one well of 2 × 10^5^ cells were summed (5 × 10^6^ cells = ten wells of 4 × 10^5^ and five wells of 2 × 10^5^ cells). An equal number of DMSO treated cells were plated as negative controls. A signal-to-noise (S:N) ratio was calculated by dividing the number of HBV-specific spots by the number spots in negative control wells treated with DMSO alone. We observed that the S:N ratio became consistent upon plating ≥ 2 × 10^6^ PBMCs (Fig. 1B). We then optimized the concentration of HBV peptides used for pulsing to maximize responses and minimize DMSO exposure. PBMCs (n = 7) were pulsed with 1, 5, and 10 µg/mL/OLP or with respective DMSO concentrations of 0.62%, 3.10% and 6.20%, with S:N ratios peaking upon pulsing with 5 µg/mL/OLP (Fig. 1C). We then re-evaluated antigen-specific responses by plating 2 × 10^6^ PBMCs for each HBV OLP pool in vaccinated donors using the optimized conditions. We observed HBV-specific T cell responses in 6/10 donors (Fig. 1D; Supplementary Fig. S1B). In addition to improving detection to 6 donors, the total number of actual counted spots increased almost tenfold in some donors. Counting more cells eliminates the need for extrapolation of spot counts, as is often done in standard ELISpot assays, and decreases assay variability. In two donors with strong responses to Env2 pool (HD19 and HD21), we observed weaker responses to Pol3. To determine if this was the result of cross-reactivity, we expanded PBMCs for 10 days with Env2 peptides and tested for responses to Env2 and Pol3. Expanded cultures responded strongly to Env2 peptides, with a weak but positive response to Pol3, indicating cross-reactivity (Supplementary Fig. S2A). Lastly, to confirm absence of non-specific responses with our stimulation strategy, we tested naïve cord blood samples (n = 5) collected from uninfected mothers and found no specific responses (Supplementary Fig. S2B). Therefore, by optimizing the variables of the ELISpot assay, we improved detection of HBV-specific T cells.

### Ex vivo* IFN-γ ELISpot responses in vaccinated donors and CHB patients*

After establishing the critical parameters for peptide pulsing, cell concentration and antigen specificity we applied the optimized ex vivo ELISpot (Fig. 2A) to measure HBV-specific T cell frequency in vaccinated donors (n = 15) and CHB patients (n = 30). Our cohort was comprised of all clinical phases of chronic HBV (Supplementary Table S3). Figure 2B,D show the absolute number of vaccinated donor and CHB patient spot forming units (SFUs) for each condition. In all subjects, the DMSO control was not significantly different than untreated cells (HD: *p* = 0.7755, CHB: *p* = 0.4966). Using a cut-off of > 25 SFU and twice the DMSO control in a total of 2 × 10^6^ PBMCs, we detected positive responses in 10/15 vaccinated donors and 20/30 CHB patients (Fig. 2C,E). Having observed an equivalent proportion of responders among vaccinated donors (67%) and CHB patients (67%), we hypothesized we reached the limit of specificity for the IFN-γ ELISpot assay. These data confirm that our adapted IFN-γ ELISpot strategy allows us to measure the ex vivo HBV-specific T cell frequency in a majority of CHB patients, which has not been demonstrated in either research or clinical studies.

### *Validation of *ex vivo* ELISpot results*

**Figure 3:**
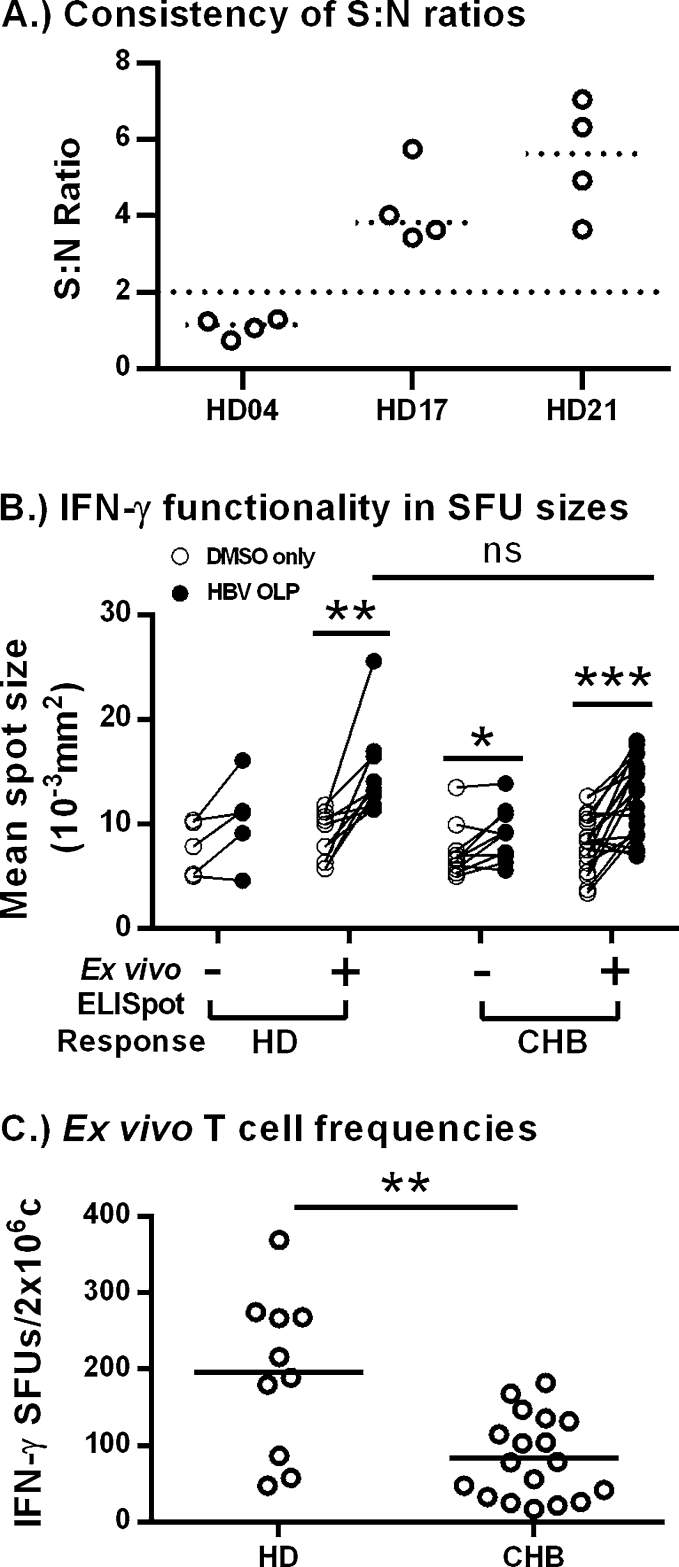
Validation and further analysis of ex vivo ELISpots. (**A**) S:N ratios of bi-weekly blood draws from vaccinated donors (n = 3) were shown to consistently indicate assay responsiveness. (**B**) Mean spot sizes were compared within each donor/patient. Levels of significance: ** p = 0.0020, * p = 0.0488, *** p = 0.0002 (Paired *t*-tests); ^n.s.^* p* = 0.1250. (**C**) SFU counts were normalized by subtracting DMSO background SFUs for vaccinated donors (n = 10) and CHB patients (n = 20). Horizontal lines indicate the mean of each group. Levels of significance: ** p = 0.0031 (Unpaired *t*-test).

Since many CHB patients displayed S:N ratios near our established positive cut-off threshold, we sought to determine the consistency of the ex vivo assay. We conducted multiple bi-weekly blood draws of 3 vaccinated donors and observed that their absolute SFUs differed from each timepoint (data not shown). However, the S:N ratios remained largely consistent; donors with a negative response remained negative and donors with a positive response remained positive when tested multiple times (Fig. 3A). We then calculated the coefficient of variation (CoV) of S:N ratios for each individual: 22.91% (HD04), 25.07% (HD17), 27.51% (HD21); averaging to 25.16%. These data indicate that a change in S:N ratio of greater than 25% could be considered as a change in the functional HBV-specific T cell response.

In addition, we analyzed IFN-γ secretion/cell by comparing control and HBV-specific spot sizes from Fig. 2B,D. Spot sizes increase as more IFN-γ is produced by a cell, representing an indirect measure of T cell functionality. SFU sizes were averaged and graphed amongst responding and non-responding vaccinated donors and CHB patients (Fig. 3B). We observed that spot sizes were significantly larger in PBMCs pulsed with HBV peptides compared to DMSO controls in both vaccinated donors (n = 10; *p* = 0.0020) and CHB patients (n = 20; *p* = 0.0002) with detectable ELISpot responses. Interestingly, HBV-OLP spot sizes between vaccinated donors and CHB patients were not significantly different (*p* = 0.3974). This suggests that T cells from CHB patients made as much IFN-γ as T cells from vaccinated donors when provided optimal stimulation. In addition, we observed increased spot sizes in HBV OLP pulsed PBMC among CHB patients that were considered negative based on our ELISpot cut-off criteria (*p* = 0.0488), suggesting we are potentially underestimating the number of CHB patients with an ex vivo T cell response. Having observed increased spot sizes in ELISpot-negative CHB patients we attempted to use spot size cut-off as a more accurate measure of reactivity in the ELISpot assay. However, despite consistent assay developing procedures, we found that changes in spot size from DMSO to HBV OLP were variable between assays and did not correlate with spot count (data not shown). Therefore, using spot size cut-offs did not improve determination of positive responses.

We then compared the number of IFN-γ producing cells between vaccinated donors (n = 10) and CHB patients (n = 20) with detectable ELISpot responses (Fig. 3C), providing absolute quantitation for the ex vivo frequency of functional HBV-specific T cells. Vaccinated donor PBMCs averaged 195 SFUs, significantly more than the average of 84 SFUs seen with CHB patient PBMCs (*p* = 0.0031). These numbers equate to a functional ex vivo frequency of 0.010% of PBMCs in vaccinated donors and 0.004% of PBMCs in CHB patients. Vaccinated donors possess two—threefold more responding HBV-specific T cells despite only responding to envelope protein contained within the prophylactic vaccine. In contrast, CHB patients have been sensitized to a larger breadth of HBV proteins and yet still display lower responses. Taken together, these data validate the ability of the optimized assay to provide consistent readouts in an assay that is known to display variability.

### *Clinical and virological parameters do not affect the *ex vivo* frequency of HBV-specific T cells*

**Figure 4:**
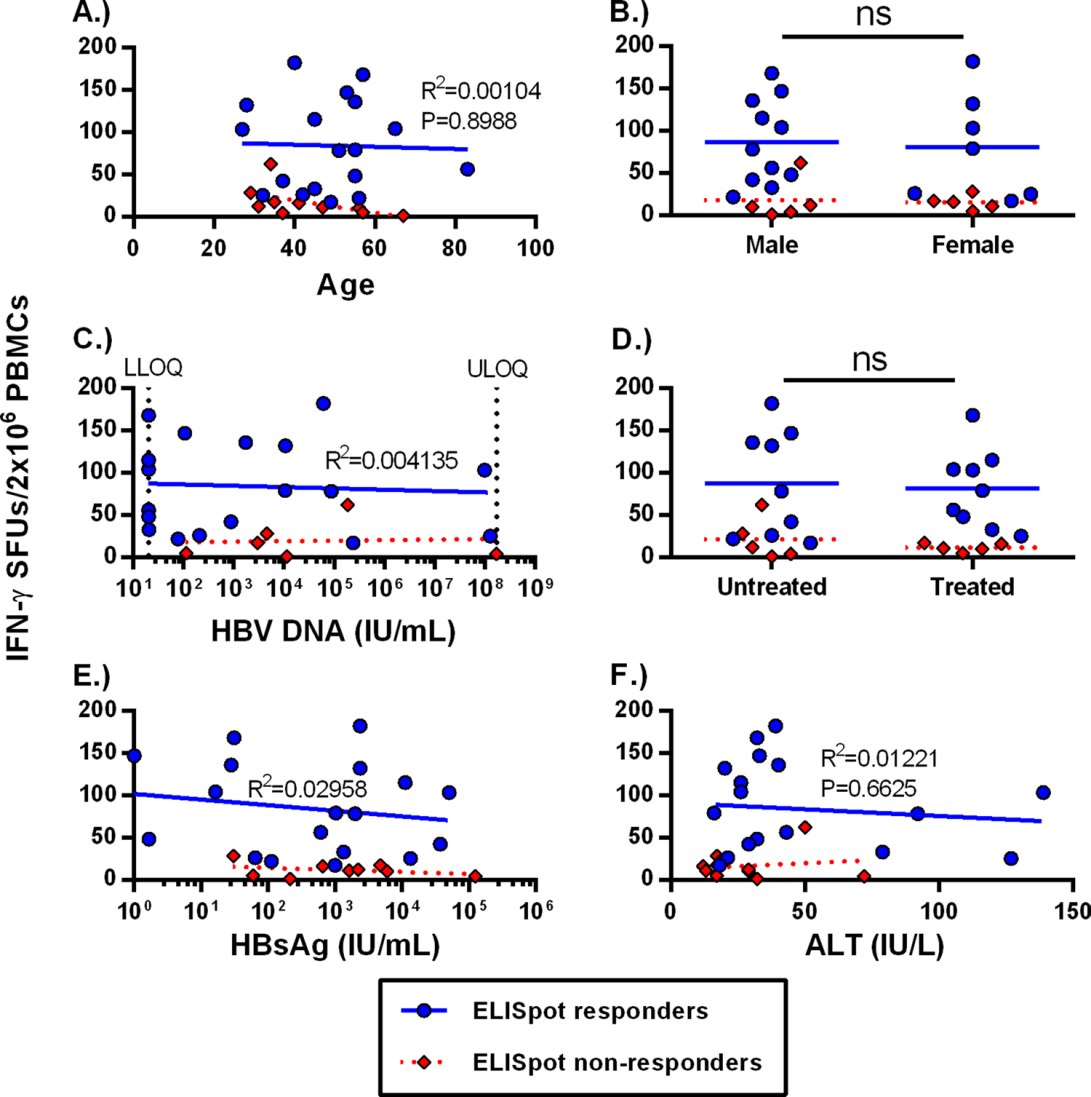
Ex vivo T cell frequencies are not altered by clinical parameters. Chronic patient HBV-specific T cell frequencies were graphed by (**A**) age (R^2^ = 0.0010), (**B**) sex (*p* = 0.6590), (**C**) HBV DNA (R^2^ = 0.0041), (**D**) treatment (*p* = 0.4153), (**E**) HBsAg levels (R^2^ = 0.0296) and (**F**) ALT (R^2^ = 0.0122). Blue circles indicate ELISpot-responding patients, while red diamonds indicate non-responders.

Recent literature suggests that CHB disease stages, which are categorized by different levels of viral load, antigen load and inflammation, do not significantly alter the HBV-specific T cell frequency^7^. However, this conclusion is based on ex vivo detection of T cells from < 20% of CHB patients. Due to the smaller sample size in our study, we categorized the 20 CHB patients with positive ex vivo ELISpot responses and compared their IFN-γ spot counts to their respective virological and clinical parameters. We categorized our responding CHB patients according to age, sex, HBV DNA levels, treatment status, HBsAg levels and ALT at the time of PBMC isolation (Fig. 4). We did not observe significant differences in total ex vivo T cell frequencies with age (Fig. 4A; R^2^ = 0.001) or sex (Fig. 4B). We did not observe correlations of T cell response with viral load (Fig. 4C; R^2^ = 0.004) or nucleoside analogue treatment status (Fig. 4D). Persistent antigen exposure during chronic infection could lead to a progressive deletion of virus-specific T cells but we did not observe any correlation with HBsAg level (Fig. 4E; R^2^ = 0.029). In addition, we found no significant correlation of HBV-specific T cell frequency with ALT levels (Fig. 4F; R^2^ = 0.01). We also did not observe significant differences based on HBeAg status; however, we had too few HBeAg + patients in our cohort (n = 4) to draw meaningful conclusions (data not shown). Importantly, the ELISpot non-responsive CHB patients (red diamonds) did not cluster with any of the parameters analyzed. These data indicate that clinical or virological parameters do not impact ex vivo detection of functional HBV-specific T cells.

### Three-color FluoroSpot improves sensitivity and measures multi-functional HBV-specific T cells

**Figure 5:**
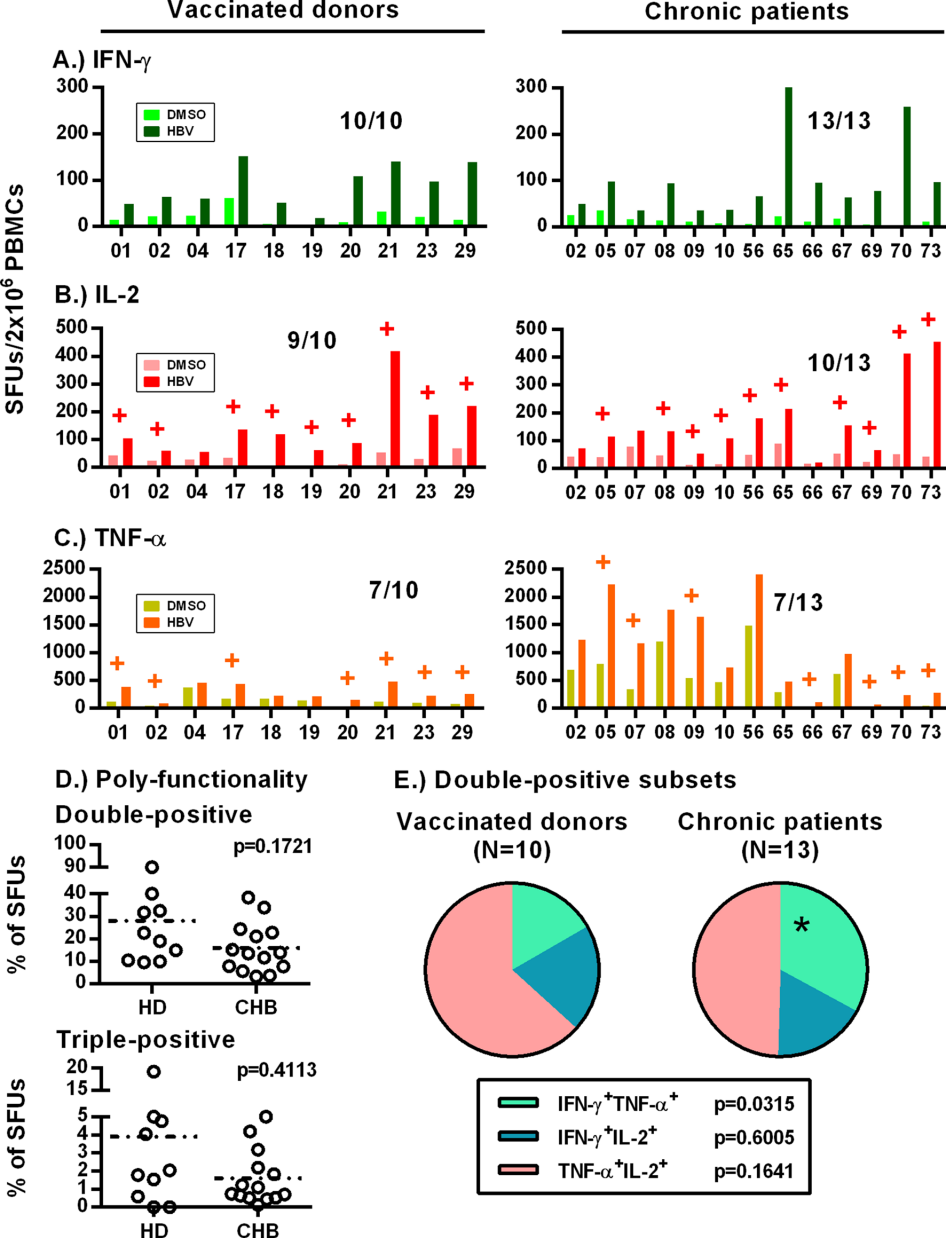
Adaptation to 3-color FluoroSpot assay improves sensitivity and measures ex vivo HBV-specific T cell multi-functionality. (**A**) IFN-γ (green), (**B**) IL-2 (red) and (**C**) TNF-α (orange) FluoroSpot responses against HBV OLP and DMSO controls. (**D**) Polyfunctionality between CHB patient and vaccinated donors were calculated as total of double-/triple-positive SFUs out of unique SFU counts. (**E**) Double-positive subsets were further delineated and compared between the two cohorts. Levels of significance are included alongside each panel where necessary.

The observation that spot sizes were significantly larger in some CHB patients with negative ELISpot responses suggested that we were underestimating the detection rate. This may be due to enzymatic amplification inherent to ELISpots, causing artificially higher background and reducing specificity. Therefore, we used the optimized ex vivo stimulation protocol to measure T cell functionality with a 3-color FluoroSpot assay measuring IFN-γ, IL-2 and TNF-α. Patient characteristics are listed online in Supplementary Table S4. FluoroSpots improved detection of IFN-γ + response in both vaccinated donors (10/10) and CHB patients (13/13; Fig. 5A). We detected IL-2 responses in 9/10 vaccinated donors and 10/13 CHB patients (Fig. 5B). TNF-α responses displayed lower sensitivity with responses detectable in 7/10 vaccinated donors and 7/13 CHB patients (Fig. 5C). While the 13 CHB patients represent a small cohort of subjects, these data suggest increased specificity of the FluoroSpot assay and a broader ex vivo functionality of HBV-specific T cells than previously realized.

We compared the frequency of multi-functional T cells in vaccinated donors and CHB patients. Vaccinated donors averaged higher frequency of T cells producing two and three cytokines compared to CHB patients, but differences did not reach statistical significance (Fig. 5D; *p* = 0.1721, *p* = 0.4113 respectively). Cells producing three cytokines were rare and did not provide sufficient numbers for robust analysis. TNF-α + IL-2 producing T cells constituted the largest proportion of T cells producing dual cytokines and were not significantly different between vaccinated donors and CHB patients. In contrast, we observed that CHB patients displayed a significant expansion of IFN-γ + TNF-α producing T cells compared to vaccinated donors (Fig. 5E; *p* = 0.0315).

A potential confounder of the increased frequency of IFN-γ + TNF-α + T cells in CHB patients was the high background observed in TNF-α. Analysis of the background TNF-α spots showed that 95.55% were single positive (n = 13), with very few TNFα + cells producing two or more cytokines in DMSO wells (Supplementary Fig. S5). However, to further assess the accuracy of this polyfunctional measurement, we tested responses to CMV, EBV and Flu (CEF) within the same patients. We found 8/10 vaccinated donors and 100% of CHB patients had detectable IFN-γ responses (Supplementary Fig. S6A), 7/10 donors and 12/13 patients had detectable IL-2 responses (Supplementary Fig. S6B) and that only 3/10 donors and 6/13 patients had detectable TNF-α responses against CEF peptides (Supplementary Fig. S6C). Double- and triple-positive T cell frequencies between donors and CHB patients were not significantly different (Supplementary Fig. S6D; *p* = 0.7844, *p* = 0.4100). Importantly, IFN-γ + TNF-α + T cell frequencies (nor any other subset) were not found to be significantly different between vaccinated donors and CHB patients upon stimulation with CEF peptides (Supplementary Fig. S6E; *p* = 0.2518). This suggests that observed differences in T cell functionality were associated with chronic HBV infection.

### HBV antigen specificity and T cell functionality among chronic patients

**Figure 6:**
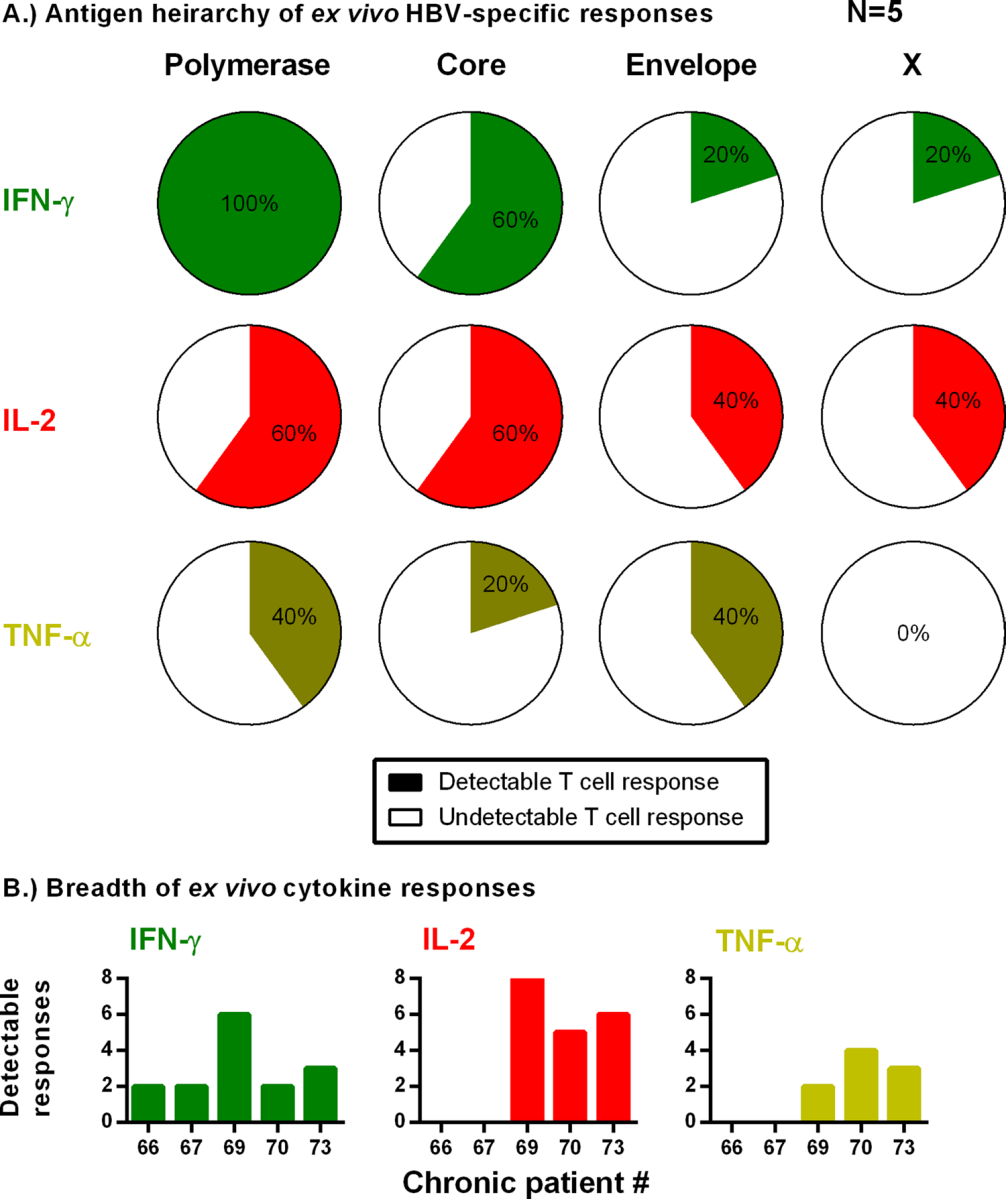
Ex vivo FluoroSpot measures antigen specific multi-functional responses in CHB patients. (**A**) Chronic patient PBMCs (n = 5; 16 × 10^6^ PBMCs per assay) were stimulated with each pool. Percentage of detectable ex vivo T cell responses (colored) against each HBV antigen (polymerase, core, envelope and X) for each cytokine are graphed. (**B**) Breadth of response for each cytokine within each tested patient. Patients could have responses to a total of 8 peptide pools (i.e. Fig. 1D).

We investigated the antigen-specific functional hierarchy of HBV-specific T cells in a small subset of CHB patients (n = 5) with sufficient PBMCs remaining to run the assay (25 × 10^6^ PBMC minimum). Patient characteristics are listed online in Supplementary Table S4. We tested multi-functional responses to individual HBV antigens, similar to vaccinated donors in Fig. 1D. Overall, IFN-γ was the dominant response with 5/5 patients responding to the polymerase pools, 3/5 to core and 1/5 each to envelope and X (Fig. 6A). IL-2 responses were detected in 3/5 patients. The IL-2 response was primarily directed towards polymerase and core; followed by envelope and X. Ex vivo IFN-γ responses were narrowly focused, with 4/5 patients responding to ≤ 3 of the 8 peptide pools (Fig. 6B). The breadth of responses in patients with IL-2 producing T cells was higher than IFN-γ, with IL-2 responses being observed to ≥ 5 peptide pools. TNF-α responses remained the least detectable, with only 2/5 patients responding towards polymerase and envelope and only one towards core. These data support previous observations that the ex vivo IFN-γ T cell response is dominated by polymerase and core and suggests CHB patients with an IL-2 response display the greatest breadth of T cell responses.

## Discussion

The absence of an assay capable of consistently measuring HBV-specific T cell functionality ex vivo in CHB patients has created a major knowledge gap in disease pathogenesis, viral control and therapeutic intervention^11,12^. In this study, we re-assessed current strategies and optimized the IFN-γ ELISpot assay to develop a protocol that can be shared and implemented in any HBV immunology laboratory. We optimized key variables of cell number, peptide concentration, and pulsing strategy, as well as validating its consistency to detect responses and its specificity towards HBV antigens. This allowed for its adaptation to the FluoroSpot assay, which further improved detection of IFN-γ + HBV-specific T cells and measurement of multi-functionality in majority of CHB patients ex vivo.

Old nomenclature of clinical stages such as “immune tolerant” have recently been challenged and our data supports that T cell responses are similar between the different stages of chronic hepatitis B^7,16,17^. However, the goal of our study was to demonstrate that HBV-specific T cells are detectable ex vivo in a majority of CHB patients and can display a functionality greater than IFN-γ production. Our cohort of 30 CHB patients were not evenly distributed by stages of disease but we did compare T cell functionality to clinical and virological parameters. We did not find any significant effect. The most robust comparision was between untreated CHB patients and patients on nucleoside analogue therapy, which has been associated with improved HBV-specific T cell expansion in vitro^5,18–21^. The lack of difference between treated and untreated patients may suggest that reduction in viral load influences the in vitro expansion capacity of HBV-specific T cells rather than their ex vivo frequency. These important questions can now be addressed using the adapted ELISpot assay.

In addition to testing T cell responses in different types of CHB patients, we used a small subset of patients to investigate antigen specificity using 3-color FluoroSpots. In the five CHB patients tested, we observed that polymerase-specific T cells dominated the ex vivo T cell response, followed by core. The retention of polymerase-specific T cells in the periphery is likely due to the lower expression of the HBV polymerase protein^8,22^. The dominance of polymerase-specific T cells may provide an explanation for the lack of differences observed between clinical and viral parameters. However, more robust analysis in different cohorts of CHB patients is required to determine if the antigen-specific hierarchy holds true in a larger cohort of patients.

In addition to antigen-specificity, we used FluoroSpots to provide evidence that HBV-specific T cells do not display an entirely exhausted functional profile ex vivo. IFN-γ was the dominant cytokine and detected in all 13 tested CHB patients. However, we detected T cells capable of producing IL-2 directly ex vivo in a high proportion of CHB patients. Patients with HBV-specific IL-2 responses showed the greatest response breadth, responding to five or more peptide pools, which is consistent with a less exhausted functional phenotype. Therefore, using the FluoroSpot assay across different patient cohorts may provide greater detail in understanding the the functional profile of HBV-specific T cells during the progression of chronic hepatitis B and the effects of therapeutic intervention.

Addressing technical issues, cell numbers are a perpetual obstacle of human research, particularly in clinical studies where intense monitoring is required. Therefore, a primary challenge was to overcome the mathematical limitation of HBV-specific T cell frequency without using a number of cells that makes the assay impractical for routine use. Standard approaches using triplicates for each HBV peptide pool (typically 8 pools) with negative and positive controls requires approximately 9 × 10^6^ PBMCs. On the other hand, our assay uses 2 × 10^6^ PBMCs/condition, simultaneously pulsed with all HBV peptides, to achieve maximum sensitivity. Incorporating the additional positive and negative controls, and cell loss due to overnight resting, the assay requires 8 × 10^6^ PBMCs. Following PBMC isolation, 10 mL of whole blood will yield 10–15 × 10^6^ PBMCs, allowing remaining cells to be analyzed with other assays from a single tube of blood; which facilitates immune monitoring in clinical trials. Therefore, we have improved sensitivity of the IFN-γ ELISpot and reduced the number of PBMCs necessary to run the assay.

A primary challenge of ELISpot is inter-assay variability and identifying a relative cut-off that accurately separates positive responses from negative responses. A cut-off of twice the background worked well in our assay but excluded some positive responses that were later shown to be detectable by FluoroSpot. We observed that majority of this variability comes from the non-specific spots detected in DMSO wells and that spot counts varied from assay-to-assay but the S:N ratio remained more consistent. To show this, we tested 3 vaccinated donors drawn at four different time points. Donors with a negative response remained negative (S:N ratio < 2) while donors with a positive response remained positive (S:N ratio ≥ 2). We tried to improve on the S:N ratio as a cut-off for positive response by comparing mean spot size between DMSO- and HBV OLP-stimulated wells. Due to the enzymatic amplification in ELISpots, absolute values for DMSO (smaller spots) or HBV-specific (larger spots) could not be standardized from experiment to experiment. Furthermore, spot sizes could not be translated to FluoroSpots because fluorescence intensity can be greatly impacted by laser intensity, exposure to light and photobleaching. Using the data from multiple time points on individual donors, we calculated the coefficient of variation (CoV) of S:N ratios to be approximately 25%. This indicates that changes in S:N ratios beyond 25% can be defined as a change in HBV-specific T cell response, allowing for longitudinal monitoring and quantification of T cell immunity among CHB patients.

In conclusion, similar to the adaptation of tetramer staining to tetramer enrichment, our adapted assay provides a new strategy to measure HBV-specific T cell immunity. Our initial use of the assay demonstrated that T cells are detectable in the majority of CHB patients ex vivo, many displaying multi-functionality. With the improved assay we, and others, can address long-standing questions regarding T cell functionality in the progression of chronic hepatitis B and the impact of novel therapies on HBV-speific T cell immunity to develop robust immunological biomarkers based on antigen-specific T cells.

## Materials and methods

### Human subjects and whole blood processing

This study was approved by the Research Ethics Board at the University Health Network and experiments were carried out in compliance with UHN guidelines. All subjects participating in the study provided written informed consent. All CHB patients were recruited at the Toronto Centre for Liver Disease. PBMC were collected by density gradient centrifugation and cryopreserved in liquid nitrogen in 90% Knockout Serum Replacement (Life Tech) + 10% DMSO (Sigma). Supplementary Table S3 summarizes clinical characteristics of CHB patients used for ELISpots in Fig. 2. Supplementary Table S4 summarizes characteristics of CHB patients used for FluoroSpots in Figs. 5 and 6 (and Supplementary Fig. S6).

### HBV overlapping peptide (OLP) pools

310 HBV genotype C, (Accession: AB112063)15-mer peptides overlapping by 10 residues, purified to > 70%, were purchased from GenScript. Peptides were dissolved in 100% DMSO to a concentration of 50 mg/mL. The 15-mer peptides were diluted in Aim-V medium and combined into 8 pools as indicated in Table 1.

**Table 1:** HBV 15-mer OLP pools.

Peptide pool	HBV region	Amino acid position	Number of peptides
1	HBV core	1–212	41
2	HBV X	1–154	29
3	HBV Env	1–181	37
4	HBV Env	186–389	36
5	HBV Pol	1–206	42
6	HBV Pol	211–416	42
7	HBV Pol	421–626	42
8	HBV Pol	631–831	41

### Ex vivo* T cell stimulation*

Cryopreserved PBMCs were thawed, resuspended in Aim-V medium (Life Tech) with 2% human serum (VWR International), and rested overnight at 37 °C in 5% CO_2_ at 4 × 10^6^ PBMCs/mL in 30 mL polypropylene tubes with caps loose. Overnight resting did not significantly alter the frequency of live cells, monocytes, total T cells, CD4 or CD8 T cells in the PBMC samples (Supplementary Fig. S7A). Cell recovery after overnight rest ranged from 60–100% and did not correlate with T cell responses (Supplementary Fig. S7B). Following rest, PBMCs were counted and 4.5 × 10^5^ PBMCs (20%) were transferred to a 1.5 mL Eppendorf tube and resuspended 84 µL Aim-V with 2% human serum. To measure the total T cell response, 2 µL of a 250 µg/mL stock of each peptide pools (Fig. 2A) were added to the cells to give a final volume of 100 µL Aim-V with a final DMSO concentration of 3.1% (v/v) and a final peptide concentration of 5 µg/mL. For individual peptide pools to test antigen specificity, 4.5 × 10^5^ PBMCs (20%) were resuspended in 98 uL Aim-V with serum in 9 separate 1.5 mL Eppendorf tubes, one for each antigenic peptide pool and the DMSO control. 2 µL of each 250 µg/mL peptide pool or DMSO vehicle was added to their respective tubes, giving a final DMSO concentration of 0.3875% (v/v) and a final peptide concentration of 5 µg/mL. Peptide loading was performed for 1 h at 37 °C in 5% CO_2_. DMSO was added as the vehicle control at equal v/v concentration as found in the peptide pool: 3.1% for the total T cell response (8 pools) or 0.39% for antigen-specific peptide pools (1 pool). After 1 h of peptide loading, PBMCs were centrifuged at 350xg, peptide loading media was removed and pulsed cells were resuspended in 225 µL Aim-V without serum. For the remaining 80% of unpulsed cells, 1.8 × 10^6^ cells were transferred to a 1.5 mL Eppendorf tube for each stimulation and centrifuged at 350xg and resuspended in 900 µL of Aim-V without serum. Peptide-loaded cells were combined with unpulsed cells in a total volume of 1.125 mL, giving a final cell concentration of 2 × 10^6^ cells/mL. Combined cells were then plated at 4 × 10^5^ PBMCs/well (200 µL) in five wells (Fig. 2A). We compared the 20:80 strategy against pulsing 100% of PBMCs with all HBV OLP pools for 1 h at 37 °C in 5% CO_2_ (Supplementary Fig. S8). The 20:80 strategy restricts DMSO exposure to a fraction of cells (20%). We found that pulsing only 20% of cells with HBV OLP maintained positive responses and resulted in lower background, providing an equal or better S:N ratio. A positive response was designated when the sum of HBV-specific SFUs from the 5 wells were both more than 25 SFUs and more than two times the background DMSO SFUs.

### IFN-γ ELISpot

96-well MultiScreenHTS-IP sterile PVDF filter plates (Merck Millipore) were activated with 35% Ethanol in ddH_2_O, washed with sterile ddH_2_O, then coated overnight at 4 °C with 5 µg/mL of mouse anti-human IFN-γ monoclonal antibody in 1 × PBS (Cellular Technologies Limited; clone: ZC-11). Capture antibody solution was discarded and the plates were washed with sterile PBS then blocked with Aim-V medium plus 10% heat-inactivated FBS for 30mins at room temperature. Blocking solution was discarded and cells were plated at 4 × 10^5^ cells/well in 5 wells, effectively plating 2 × 10^6^ cells/condition. Wells containing PBMCs with human T-activator CD3/28 beads (Thermo Fischer Scientific) or without any stimulation or DMSO were used as positive and negative controls (7.5 × 10^4^ and 1 × 10^6^ cells respectively). Plates were incubated at 37 °C in 5% CO_2_ for 24 h. Following the incubation, cells were discarded and plates were washed with PBS. Biotinylated mouse anti-human IFN-γ monoclonal antibody (Cellular Technologies Limited; clone: ZD-51) solution was added to each well at 0.5 µg/mL for 2 h at room temperature, discarded and washed. Streptavidin-ALP was added to each well at 0.25 ng/mL for 30mins at room temperature, discarded and washed. A substrate solution of 330 µg/mL NBT and 165 µg/mL BCIP (Promega) was prepared in alkaline phosphatase buffer (100 mM Tris–HCl [pH 9.0], 150 mM NaCl, 1 mM MgCl_2_), and 50 µL were added to each well for 20mins in the dark. Substrate solution was discarded and plates were washed with distilled water before air drying. Spot-forming units were counted using the C.T.L. ImmunoSpot S6 Analyzer (Cellular Technology Limited).

### 3-Color FluoroSpot

FluoroSpot kits (Cellular Technologies Ltd.) detecting IFN-γ (green), IL-2 (red), and TNF-α (yellow) secretion were coated and developed as per manufacturer’s protocol. Ex vivo PBMC stimulation and plating was carried out as described above to measure HBV-specific T cell responses.

To measure antigen specific T cell responses with the 8 peptide pools covering each HBV protein, 2 × 10^6^ PBMCs were pulsed with 5 µg/mL peptide for each peptide pool (Table 1) using the protocol described for total HBV T cell responses (20% of cells loaded with peptide). 1 × 10^6^ PBMCs stimulated with 33 known epitopes targeting cytomegalovirus, Epstein Barr virus and influenza virus (CEF, GenScript; 1 µg/mL/peptide) and 7.5 × 10^4^ PBMCs stimulated with human T-activator CD3/28 beads were used as positive controls. A positive response is designated when there were more than 25 SFUs and more than two times the background DMSO SFUs.

### Statistical analysis

Wilcoxon tests were conducted between untreated and DMSO control SFU counts in HDs and CHB patients. Wilcoxon tests were conducted to compare DMSO and OLP SFU sizes in HDs and CHBs (Fig. 3B); while a Mann–Whitney test was performed between responding HD and CHB OLP SFUs. Mann–Whitney tests were conducted to compare normalized HD and CHB SFUs (Fig. 3C); and to compare SFU counts between sex, and treatment status (Fig. 4B, D). Regression lines were displayed with R-squared calculated for SFU counts against age, HBV DNA, serum HBsAg and ALT levels (Fig. 4A,C,E,F). Mann–Whitney tests were conducted to compare polyfunctionality (Fig. 5D, Supplementary Fig. S6D) and double-positive subsets between HDs and CHBs (Fig. 5E, Supplementary Fig. S6E).

## Supplementary information

Supplementary file1
