# Supplementary material for: Optimized ex vivo stimulation identifies multi-functional HBV-specific T cells in a majority of chronic hepatitis B patients
Source: Sci Rep. 2020 Jul 9;10:11344. doi: 10.1038/s41598-020-68226-5 (PMC7347526; doi:10.1038/s41598-020-68226-5)
Supplement: Supplementary file 1 — Supplementary file1 [file 41598_2020_68226_MOESM1_ESM.docx]

**Optimized Ex Vivo Stimulation Identifies Multi-functional HBV-specific T cells in a Majority of Chronic Hepatitis B Patients**

Conan G. Chua^1,2^, Aman Mehrotra^1^, Tony Mazzulli^3,4^, David K.H. Wong^1^, Jordan J. Feld^1^, Harry L.A. Janssen^1^, Adam J. Gehring^1,2^

^1^Toronto Centre for Liver Disease, Toronto General Research Institute, University Health Network, Toronto, Canada

^2^Institute of Medical Sciences, University of Toronto, Toronto, Canada

^3^Department of Laboratory Medicine and Pathobiology, University of Toronto, Toronto, Canada

^4^Department of Microbiology, Mount Sinai Hospital, University Health Network, Toronto, Canada

**Corresponding author:**

Adam J. Gehring, Ph.D.

Toronto General Hospital Research Institute

Princess Margaret Cancer Research Tower

Room 10-356

101 College St,

Toronto, ON M5G 1L7

Canada

Tel: +1 (416) 634-7095

Email: adam.gehring@uhnresearch.ca


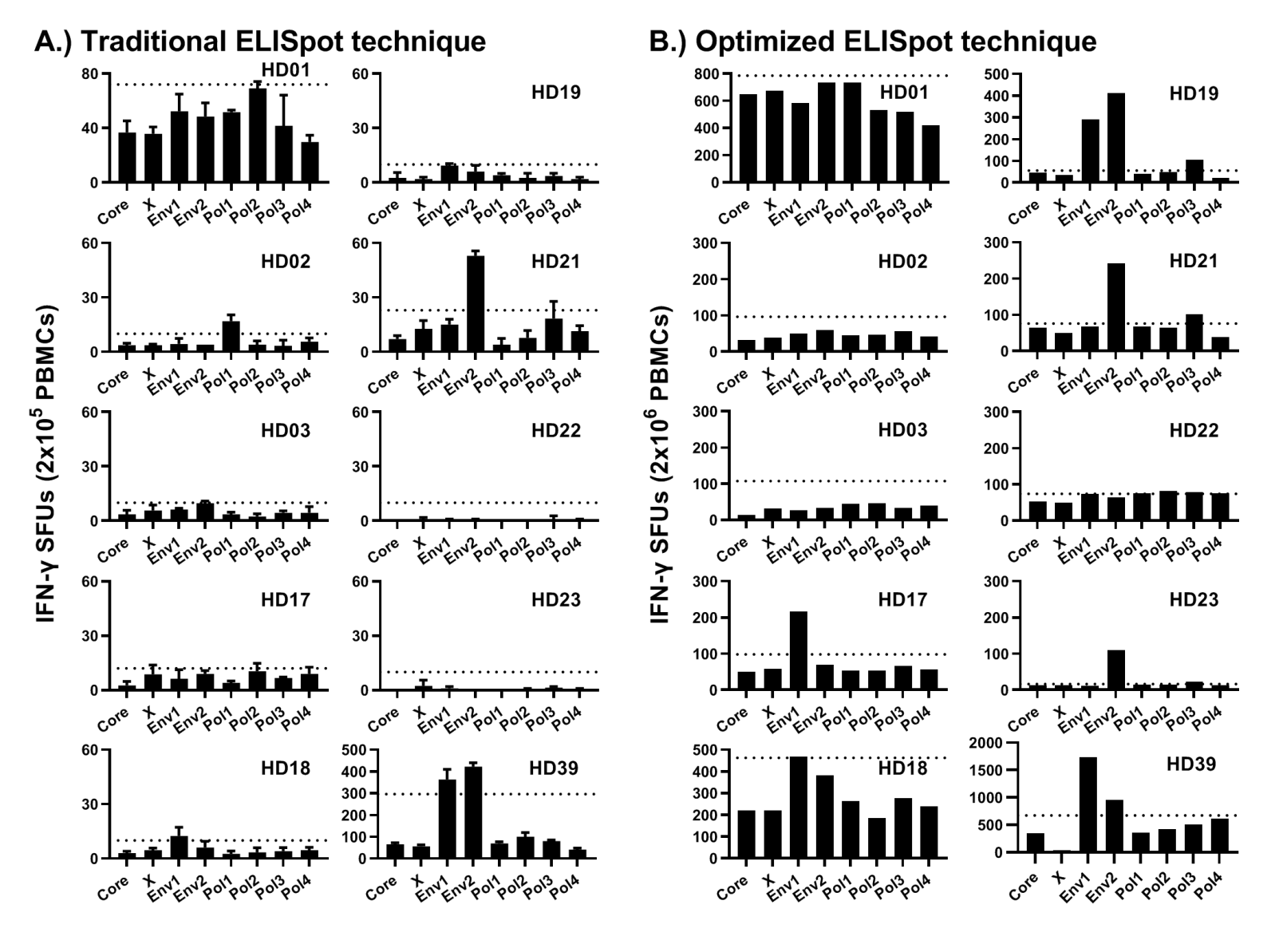


**Supplementary Figure S1:**

**Supplementary Figure S1. Comparison of traditional and optimized *ex vivo* ELISpot protocols.** Vaccinated donor PBMCs were thawed and rested overnight at 37^o^C. (**A**) 2x10^5^ PBMCs were plated in triplicate and stimulated overnight with 5µg/mL/OLP of each pool. Results per pool were averaged over triplicates with error bars; dotted lines indicate 2x DMSO SFUs or a cut-off of 10 SFUs. (**B**) 4.5x10^5^ PBMCs (20%) were pulsed with 5µg/mL/OLP of each pool for 1 hour at 37^o^C. Cells were washed then pooled with 1.8x10^6^ PBMCs (80%). Results per pool were summed; dotted lines indicate 2x DMSO SFUs or a cut-off of 25 SFUs.


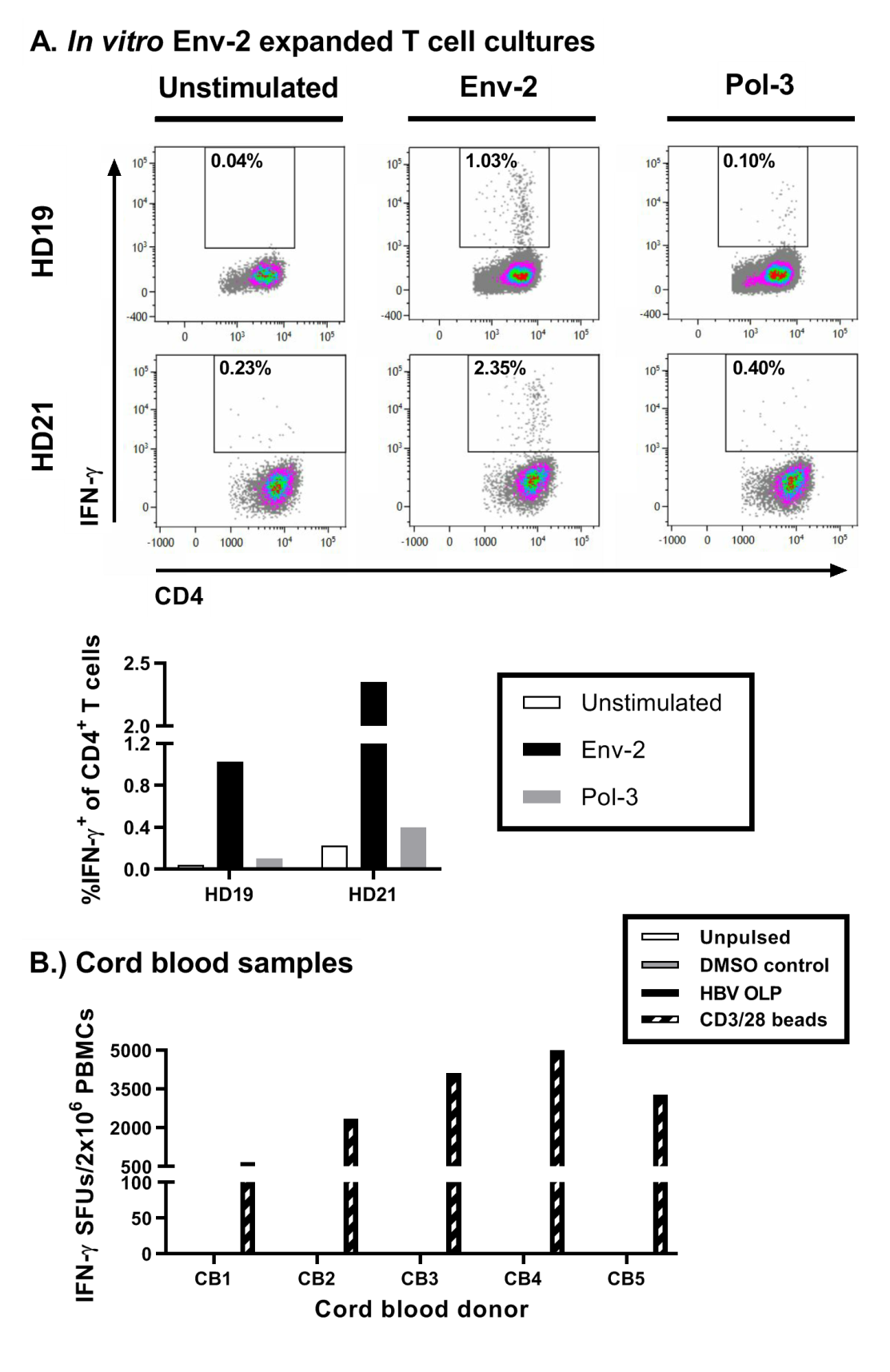


**Supplementary Figure S2. *Ex vivo* peptide stimulation does not induce non-specific T cell responses. (A)** Env-2 pool stimulated PBMCs were expanded *in vitro* with 10µg/mL IL-2 for 7 days. Expanded PBMCs were re-stimulated with either Env-2 or Pol-3 pools (or unstimulated) and 10µg/mL BFA. Cells were stained and prepared for FACS analysis. **(B)** Human cord blood mononuclear cells (CBMCs) from 5 donors were stimulated as previously described. No IFN-γ SFUs were detected from HBV OLP or DMSO stimulation. SFUs from CD3/28 bead stimulation demonstrated T cell responsiveness of each sample. Results were extrapolated from 50k CBMCs.

**Supplementary Figure S2:**

**Supplementary Table S3:**

| **Patient ID** | **Sex** | **Age** | **HBeAg Status** | **HBVsAg (IU/ml)** | **ALT (IU/L)** | **HBV DNA (IU/mL)** | **Clinical phase** | **Treatment** | **ELISpot response** |
| --- | --- | --- | --- | --- | --- | --- | --- | --- | --- |
| CHB007 | F | 47 | (-) | 4825.72 | 13 | 0.00 | On treatment | Treated | (-) |
| CHB008 | F | 42 | (-) | 64.48 | 21 | 207.00 | Inactive | Untreated | (+) |
| CHB011 | F | 55 | (-) | 1037.04 | 16 | 10400.00 | On treatment | Treated | (+) |
| CHB016 | F | 32 | (+) | 13447.10 | 127 | 127000000.00 | On treatment | Treated | (+) |
| CHB017 | F | 29 | (-) | 5983.23 | 17 | 4620.00 | Indeterminate | Untreated | (-) |
| CHB020 | F | 57 | (-) | 30.38 | 17 | 112.00 | Inactive | Treated | (-) |
| CHB021 | F | 41 | (-) | 1626.84 | 12 | 0.00 | Inactive | Treated | (-) |
| CHB025 | F | 35 | (-) | 659.18 | 17 | 3010.00 | Inactive | Treated | (-) |
| CHB026 | M | 65 | (-) | 16.51 | 26 | 0.00 | On treatment | Treated | (+) |
| CHB027 | M | 51 | (-) | 2000.95 | 92 | 87000.00 | Immune-active | Untreated | (+) |
| CHB028 | M | 41 | (-) | 4019.70 | 53 | 182000.00 | Immune-active | Untreated | (+) |
| CHB030 | M | 56 | (-) | 111.77 | 19 | 76.60 | Inactive | Untreated | (+) |
| CHB031 | M | 56 | (-) | 60.23 | 29 | 0.00 | On treatment | Treated | (-) |
| CHB033 | M | 54 | (-) | 0.76 | 33 | 106.00 | Inactive | Untreated | (+) |
| CHB034 | F | 40 | (-) | 2396.02 | 39 | 61000.00 | Immune-active | Untreated | (+) |
| CHB035 | F | 27 | (+) | 51229 | 139 | 98000000.00 | On treatment | Treated | (+) |
| CHB036 | F | 49 | (+) | 1011.22 | 18 | 238000.00 | Immune-tolerant | Untreated | (+) |
| CHB037 | M | 57 | (-) | 31.27 | 32 | 0.00 | On treatment | Treated | (+) |
| **Patient ID** | **Sex** | **Age** | **HBeAg** | **HBVsAg (IU/ml)** | **ALT (IU/L)** | **HBV DNA (IU/mL)** | **Clinical phase** | **Treatment** | **ELISpot response** |
| CHB038 | M | 67 | (-) | 212.73 | 32 | 11600.0 | Indeterminate | Untreated | (-) |
| CHB040 | M | 84 | (-) | 616.37 | 43 | 0.0 | On treatment | Treated | (+) |
| CHB041 | M | 55 | (-) | 1.66 | 32 | 0.0 | On treatment | Treated | (+) |
| CHB042 | M | 55 | (-) | 28.36 | 40 | 1720.0 | Inactive | Untreated | (+) |
| CHB043 | M | 45 | (-) | 1357 | 79 | 20.4 | On treatment | Treated | (+) |
| CHB044 | M | 45 | (-) | 95.38 | 49 | 76.3 | On treatment | Treated | (+) |
| CHB046 | F | 28 | (+) | 2395.55 | 20 | 10700.0 | Immune-tolerant | Untreated | (+) |
| CHB047 | M | 37 | (+) | >124925.00 | 72 | 170000000.0 | Immune-active | Untreated | (-) |
| CHB048 | M | 37 | (-) | 37606 | 29 | 878.0 | Indeterminate | Untreated | (+) |
| CHB049 | M | 31 | (-) | 0.00 | 33 | 0.0 | Inactive | Untreated | (-) |
| CHB060 | M | 34 | (-) | 2227.8 | 50 | 185000.0 | Immune-active | Untreated | (-) |
| CHB061 | M | 46 | (-) | 11190.77 | 26 | 0.0 | On treatment | Treated | (+) |

**Supplementary Table S3.** Patient characteristics for *ex vivo* ELISpots (Fig. 2). Patient clinical phases are determined according to 2018 AASLD guidelines or are classified as ‘indeterminate’ otherwise.

**Supplementary Table S4:**

| **Patient ID** | **Sex** | **Age** | **HBeAg** | **HBVsAg (IU/mL)** | **ALT (IU/L)** | **HBV DNA (IU/mL)** | **Clincal phase** | **Treatment** | **IFN-γ** | **TNF-α** | **IL-2** |
| --- | --- | --- | --- | --- | --- | --- | --- | --- | --- | --- | --- |
| CHB002 | M | 49 | (-) | 1518.00 | 25 | 4250.0 | Indeterminate | Untreated | (+) | (-) | (-) |
| CHB005 | M | 29 | (+) | 108981.00 | 47 | 170000000.0 | Immune tolerant | Untreated | (+) | (+) | (+) |
| CHB007 | F | 45 | (-) | 4825.72 | 13 | 0.0 | On treatment | Treated | (+) | (+) | (-) |
| CHB008 | F | 41 | (-) | 64.48 | 21 | 207.0 | Inactive | Untreated | (+) | (-) | (+) |
| CHB009 | M | 49 | (+) | 21215.70 | 275 | 185000.0 | On treatment | Treated | (+) | (+) | (+) |
| CHB010 | F | 42 | (+) | 7147.00 | 52 | 170000000.0 | On treatment | Treated | (+) | (-) | (+) |
| CHB056 | M | 49 | (-) | 26.72 | 54 | 0.0 | Inactive | Untreated | (+) | (-) | (+) |
| CHB065 | F | 54 | (-) | 5.60 | 25 | 199.0 | Inactive | Untreated | (+) | (-) | (+) |
| CHB066 | M | 53 | (-) | 1005.03 | 25 | 0.0 | On treatment | Treated | (+) | (+) | (-) |
| CHB067 | M | 69 | (-) | 95.10 | 20 | 0.0 | On treatment | Treated | (+) | (-) | (+) |
| CHB069 | F | 33 | (-) | 4657.55 | 19 | 0.0 | On treatment | Treated | (+) | (+) | (+) |
| CHB070 | F | 43 | (-) | 26.94 | 17 | 666.0 | Inactive | Untreated | (+) | (+) | (+) |
| CHB073 | M | 43 | (-) | 1116.35 | 31 | 0.0 | On treatment | Treated | (+) | (+) | (+) |

**Supplementary Table S4.** Patient characteristics for *ex vivo* FluoroSpots (Figs. 5 and 6). Patient clinical phases are determined according to 2018 AASLD guidelines or are classified as ‘indeterminate’ otherwise.


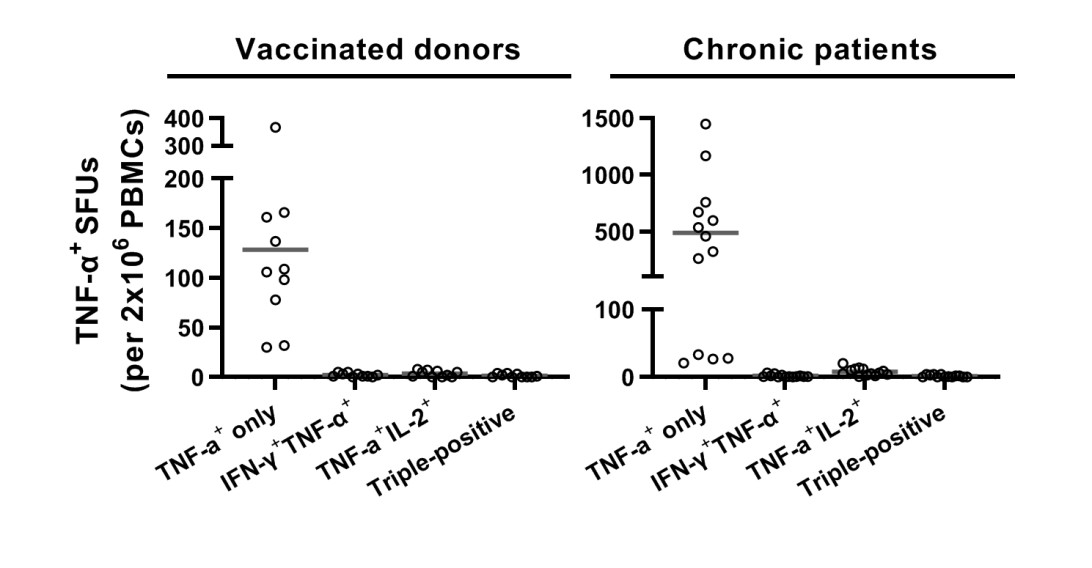


**Supplementary Figure S5:**

**Supplementary Figure S5. Majority of TNF-α SFUs comprise of single-positive cells.** TNF-α responses from DMSO-stimulated PBMCs in figure 5 were analyzed for functionality. Majority (>90%) are found to be single-positive for TNF-α^+^ secretion only.

**Supplementary Figure S6:**


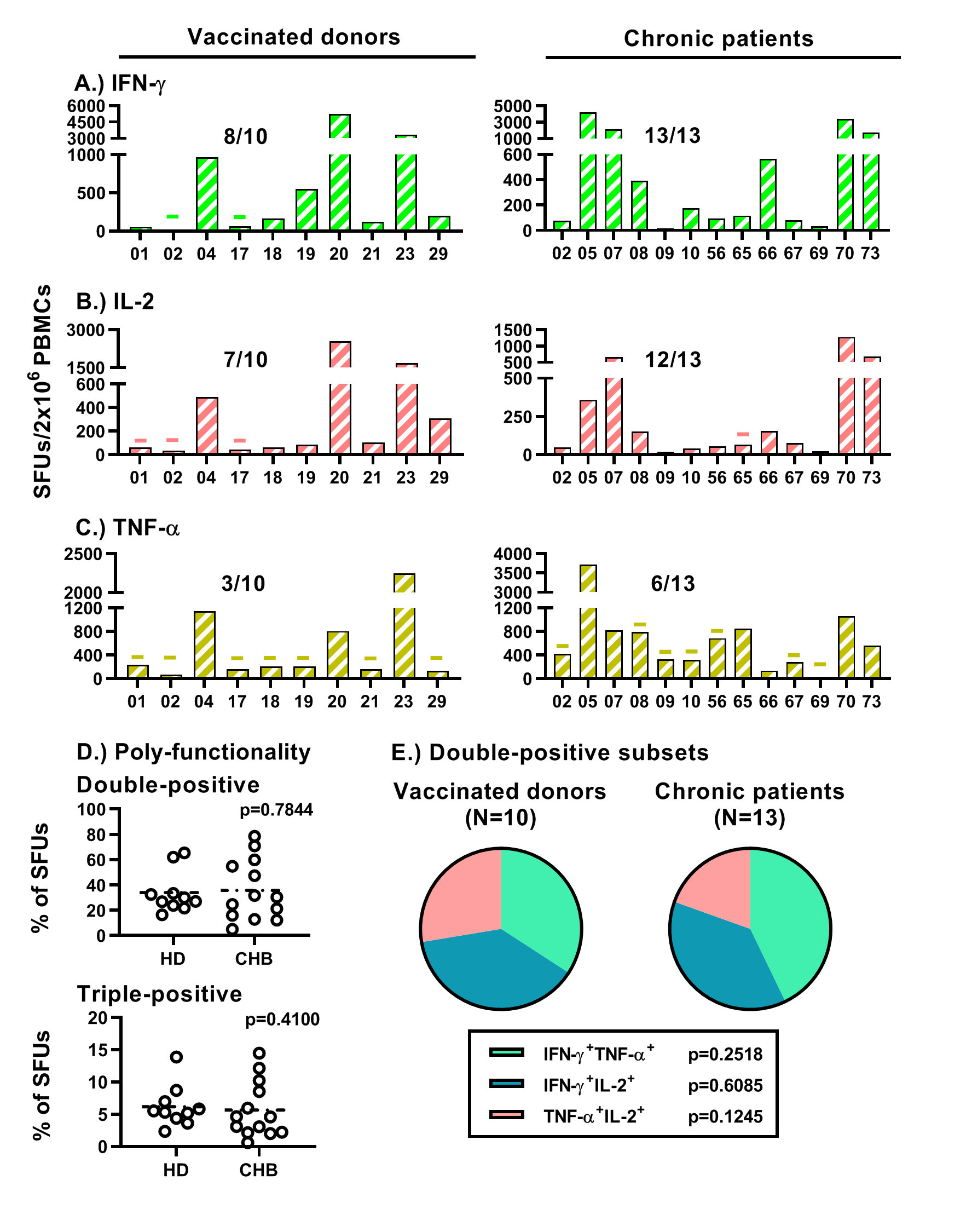


**Supplementary Figure S6. Functionality of CEF-stimulated PBMCs. (A)** IFN-γ (green), **(B)** IL-2 (red) and **(C)** TNF-α (orange) FluoroSpot responses against CEF peptides. **(D)** Polyfunctionality between CHB patient and vaccinated donors were calculated as total of double-/triple-positive SFUs out of unique SFU counts. **(E)** Double-positive subsets were further delineated and compared between the two cohorts. Levels of significance are included alongside each panel where necessary.


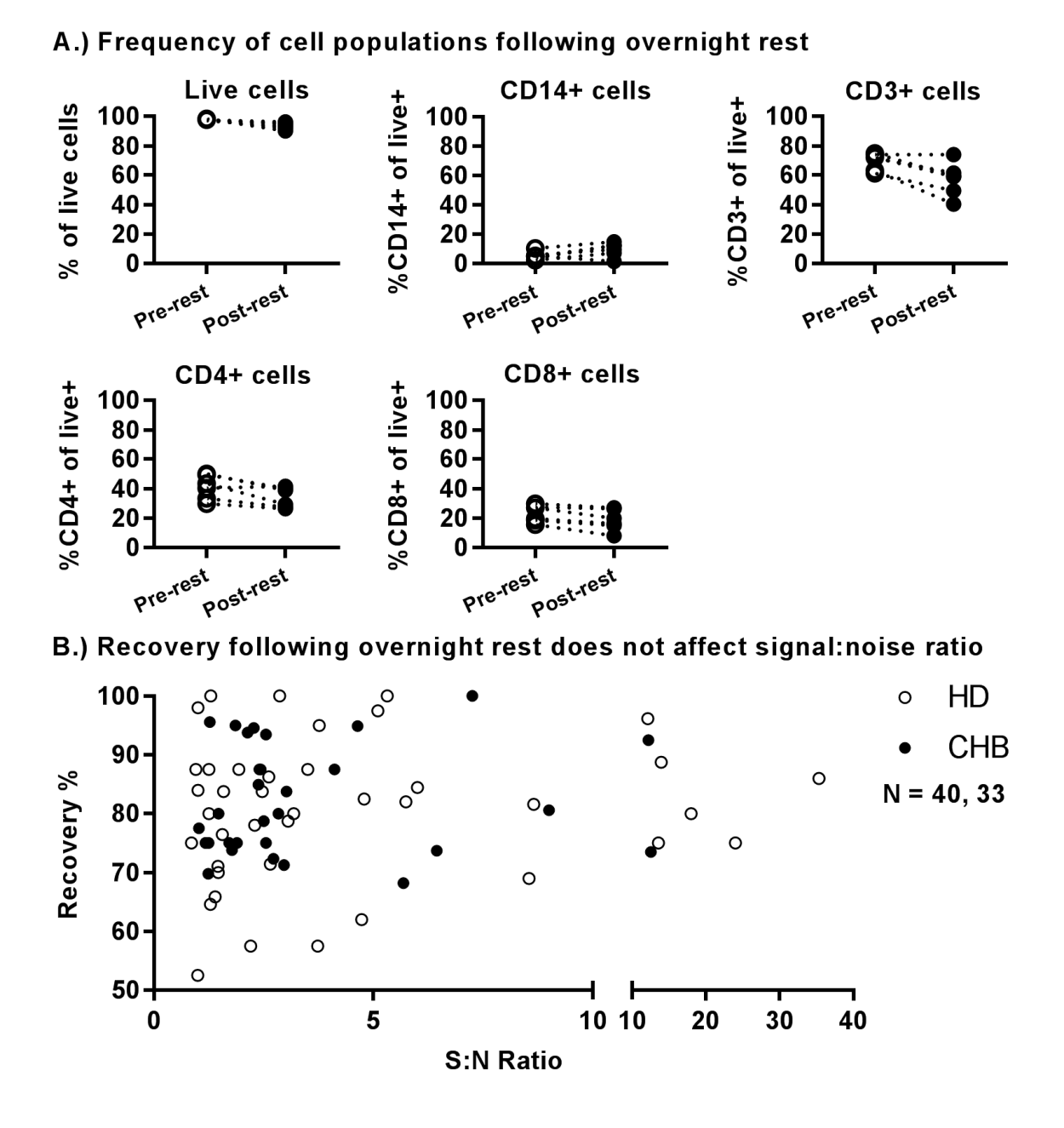


**Supplementary Figure S7. Overnight resting does not significantly affect immune cell populations or *ex vivo* assay readout. (A)** Frequencies of live cells, monocytes (CD14^+^), and T cells (CD3^+^CD4^+^ or CD3^+^CD8^+^) before and after overnight rest. **(B)** Recovery following overnight rest graphed against *ex vivo* ELISpot signal:noise ratio for HDs and CHBs.

**Supplementary Figure S7:**


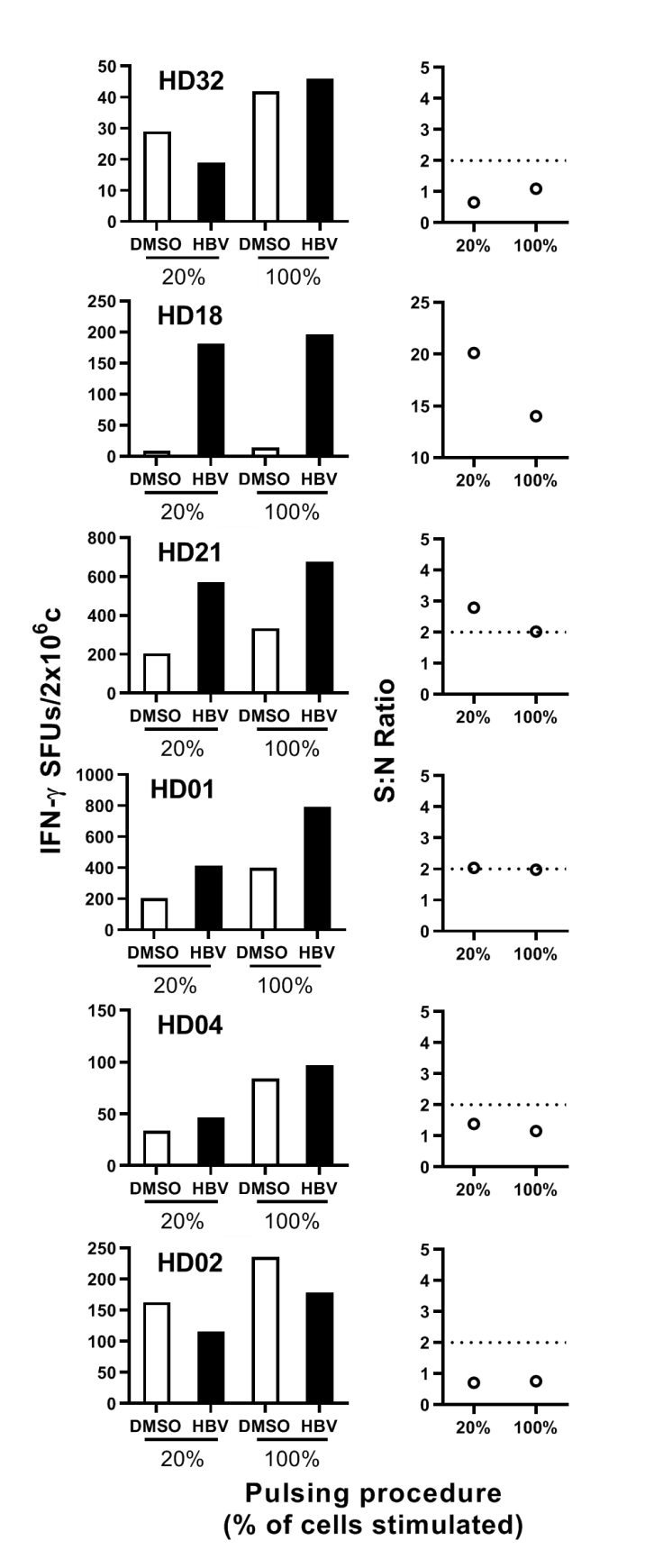


**Supplementary Figure S8:**

**Supplementary Figure S8. 20:80 pulsing strategy results in less DMSO background.** 20% and 100% of vaccinated donor PBMCs (N=6) were exposed to OLP/DMSO for 1 hour at 37^o^C. 20% pulsed cells were pooled with remaining unpulsed 80% of cells following incubation. Cells were plated on IFN-γ ELISpot plates and S:N ratios were calculated.
